# Supplementary material for: Role of LIN28B in the Regulation of Ribosomal Biogenesis and Lipid Metabolism in Medulloblastoma Brain Cancer Cells
Source: Proteomes. 2025 Mar 27;13(2):14. doi: 10.3390/proteomes13020014 (PMC12015845; doi:10.3390/proteomes13020014)

Role of LIN28B in the regulation of ribosomal biogenesis and lipid metabolism in medulloblastoma brain cancer cells

Ahmed Maklad, Mohammed Sedeeq, Kaveh Baghaei, Richard Wilson, Nuri Gueven, Iman Azimi

Original Western Blot images

Unprocessed Western Blot images for Figure 1A

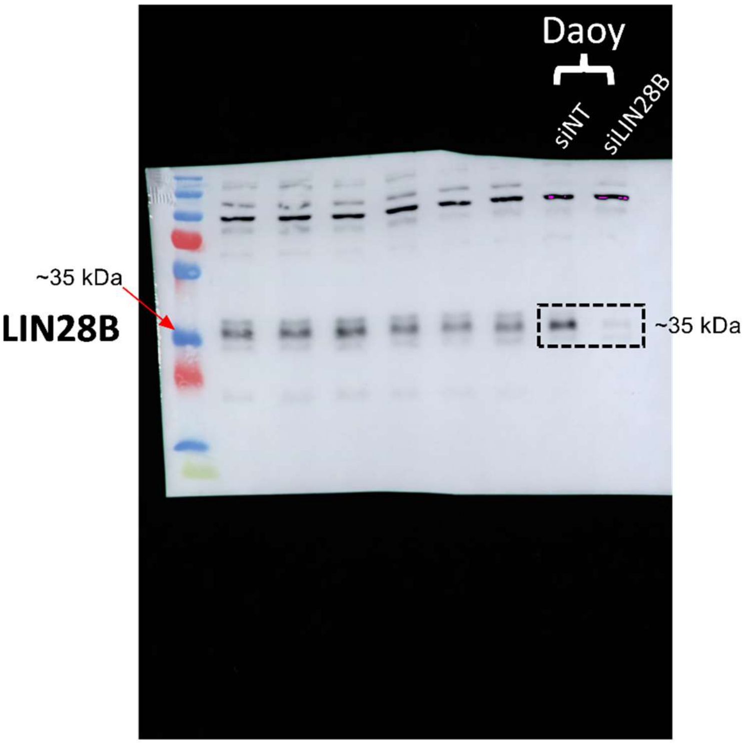

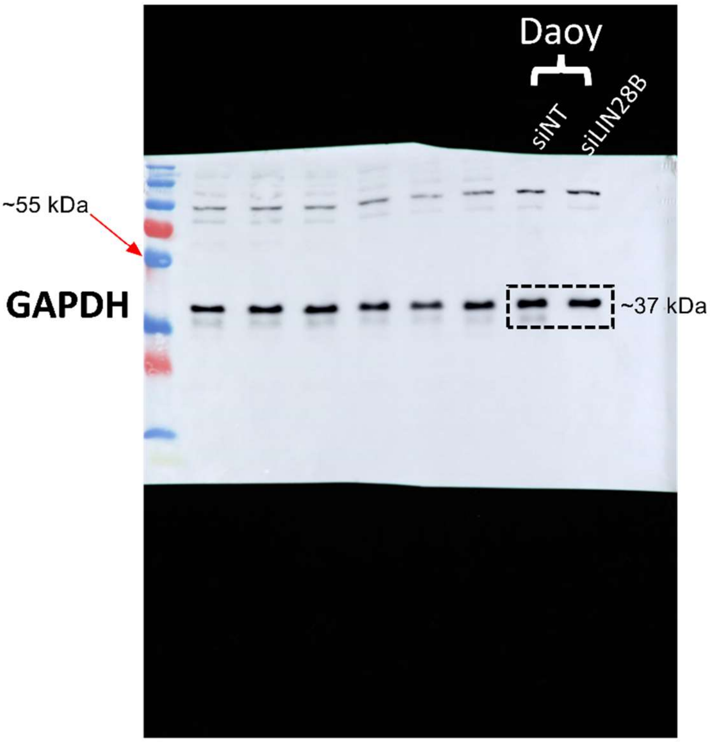

Unprocessed Western Blot images for Figure 3D

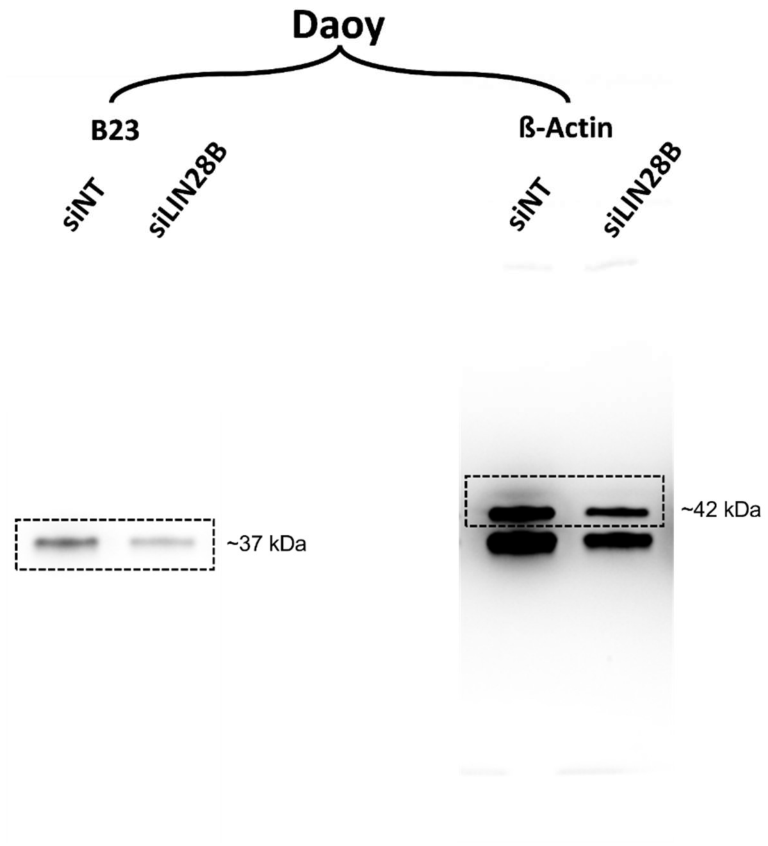

Lower contrast, allowing visualisation of the ladder in Lane 1

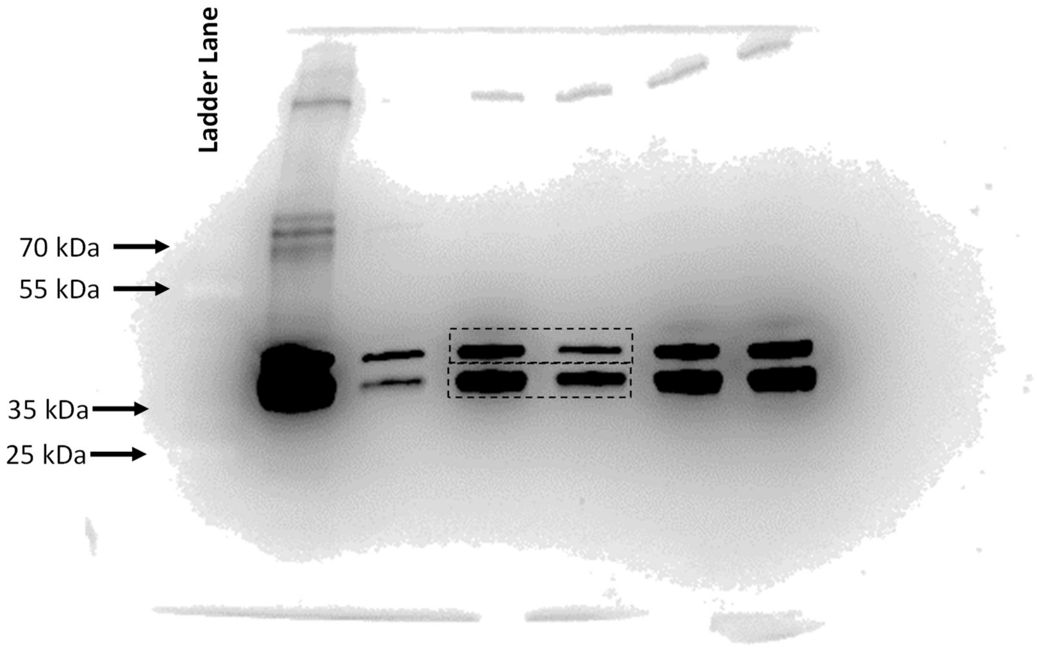

Supplement: Supplementary file 1 [file proteomes-13-00014-s001.zip › proteomes-3469515-Original Images for Western Blot.pdf]
